# Mechanical homeostasis regulating adipose tissue volume

**DOI:** 10.1186/1746-160X-3-34

**Published:** 2007-09-24

**Authors:** Paul Svedman

**Affiliations:** 1 Department of Plastic and Reconstructive Surgery, Lund University, University Hospital MAS, SE-20502, Malmö, Sweden

## Abstract

**Background:**

The total body adipose tissue volume is regulated by hormonal, nutritional, paracrine, neuronal and genetic control signals, as well as components of cell-cell or cell-matrix interactions. There are no known locally acting homeostatic mechanisms by which growing adipose tissue might adapt its volume.

**Presentation of the hypothesis:**

Mechanosensitivity has been demonstrated by mesenchymal cells in tissue culture. Adipocyte differentiation has been shown to be inhibited by stretching in vitro, and a pathway for the response has been elucidated. In humans, intermittent stretching of skin for reconstructional purposes leads to thinning of adipose tissue and thickening of epidermis – findings matching those observed in vitro in response to mechanical stimuli. Furthermore, protracted suspension of one leg increases the intermuscular adipose tissue volume of the limb. These findings may indicate a local homeostatic adipose tissue volume-regulating mechanism based on movement-induced reduction of adipocyte differentiation. This function might, during evolution, have been of importance in confined spaces, where overgrowth of adipose tissue could lead to functional disturbance, as for instance in the turtle. In humans, adipose tissue near muscle might in particular be affected, for instance intermuscularly, extraperitoneally and epicardially. Mechanical homeostasis might also contribute to protracted maintainment of soft tissue shape in the face and neck region.

**Testing of the hypothesis:**

Assessment of messenger RNA-expression of human adipocytes following activity in adjacent muscle is planned, and study of biochemical and volumetric adipose tissue changes in man are proposed.

**Implications of the hypothesis:**

The interpretation of metabolic disturbances by means of adipose tissue might be influenced. Possible applications in the head and neck were discussed.

## Background

The total body adipose tissue volume may be determined by the rate of adipocyte formation, the number of adipocytes and their average volume. Increases in adipocyte number occur via differentiation of preadipocytes [1], and decreases via preadipocyte and adipocyte apoptosis. This regulation is complex and involves hormonal, nutritional, paracrine and neuronal control signals, as well as components of cell-cell or cell-matrix interactions [2]. Evidence for a genetic influence is forthcoming [3]. The development of obesity is dependent on an interplay between adipocyte hypertrophy, adipocyte hyperplasia and angiogenesis [2].

There are no known locally acting homeostatic mechanisms by which growing adipose tissue might adapt its volume, in particular in confined spaces in the body where its unimpeded growth might lead to functional disturbance.

## Presentation of the hypothesis

The assumption of locally controlled, mechanical homeostasis of adipose tissue is indirectly supported by the large body of evidence accumulated from studies of different types of mesodermal cells in culture [4], which show that mechanical stimuli (stretch, pressure, traction and shear) cause direct stretching of protein-cell surface integrin binding sites in the plasma membrane, and by findings showing that changes in the local chemistry of the extracellular matrix induced by local movement may alter integrin structure and lead to activation of secondary messenger pathways within the cell. This activation leads to altered regulation of genes controlling break-down and formation of extracellular matrix proteins, as well as to alterations in cell division, i.e. new cell growth or apoptosis.

Adipocyte maturation may be followed from preadipocyte precursor cells in cell culture models. While such models are widely used for elucidating cellular and biochemical pathways in obesity [2], direct effects of mechanical stimulation on adipocyte differentiation have been reported upon in only one study [5]. In this work, a model was used in which a culture of preadipocyte 3T3-L1 mouse cells, growing on a silicone membrane, was stretched cyclically and longitudinally at 1 Hz during an induction period of 45 h, with the degree of stretch ranging from 130% to 175% of the initial length. This was followed by a cell maturation period lasting 9 days. During the induction the cells became deformed and tended to orient uni-directionally. Adipocyte differentiation was inhibited, but only by mechanical stimulation during the final part of the period. It was shown that the apoptotic stimulus was mainly attributable to a reduced expression of adipogenic transcription factor peroxisome proliferator-activated receptor (PPAR)gamma(2) via the activation of an extracellular signal-regulated protein kinase (ERK/MAPK) pathway [5,6]. It has been suggested that stimulation of this pathway might have opposing effects in the process of adipogenesis, depending on the time of activation during the differentiation process [7]. According to Tanabe et al., there was no sign of this in their study [5], where only the inhibitory role of the ERK/MAPK pathway was elicited. Based on these findings the authors suggested that mechanical stimuli might act analogously in humans, and massage or mechanical vibration were tentatively proposed for reducing adipose tissue locally. Mechanical stretcing of human epidermal keratinocytes studied in vitro results instead in cell proliferation, and a pathway for this response has also been clarified [8,9].

In reconstructive surgery a technique is used in which tissue defects too large to be directly sutured may be reconstructed by stretching the adjacent soft tissue at intervals from below for weeks or months by means of a series of injections of fluid into an implanted balloon. Once the area of the stretched tissue matches the defect, the balloon is removed and the surplus tissue is surgically transferred into the defect. The thickness of the subcutaneous adipose tissue overlying the balloon has been found to be decreased by the stretching (56% reduction from pre-test value at full expansion) [10], while the thickness of the epidermis became increased [11]. These changes – recorded in compartments where cell-cell interactions predominate – are in accordance with findings in cell culture models of the respective cells [5,8], and the matching responses to mechanical stimuli in vitro and in vivo strengthen the validity of the clinical findings.

Further support for a mechanical homeostasis may derive from a study in young adults in which the volume of subfascial intermuscular adipose tissue (IMAT)[12] in one lower limb was measured by magnetic resonance imaging following an initial 4 week control period of normal physical activity, and then following 4 weeks during which leg ground contact was eliminated by elevating the sole of the shoe on the contralateral side [13]. IMAT was increased by 15–20% by this activity restriction, while the volume of subcutis remained unchanged and the volunteers lost 1.3% of the body weight. The increase in IMAT was explained by an excessive triacylglycerolinflux from the vasculature or higher levels of glucolytic muscle metabolism causing dysregulation of intramyocellular lipids. A complementary mechanism may be that part of the increase in the IMAT volume was caused by the absence of regular muscular movement inducing adipocyte apoptosis.

The proposed function might provide a dynamic balance between the rate of adipocyte formation, the maintainment of a certain volume of adipose cells in a particular anatomical space, and the rate of apoptosis. The function would be of most evident importance in relatively confined spaces in the body where unimpeded adipose tissue growth might lead to functional disturbance or ischemia. Here it might serve as an "overflow" control preventing compression – of particular value in situations where external expansion may be limited by exoskeleton or relatively dense dermis, as for instance in the turtle. Subfascial spaces may constitute another example. During the course of evolution, functional benefit might thus have supported the expression of such a mechanism.

In humans, adipose tissue near muscle might in particular be affected, for instance intermuscularly, extraperitoneally and epicardially. The adipose tissue adjacent to the subcutaneous face and neck muscles may constitute a special case of intermuscular distribution, as may the orbital cavity where adipose tissue surrounds the ocular muscles and is limited anteriorly by the eye bulb, the orbital septum and the orbicular muscle of the eyelid. The mechanical stimuli causing adipose apoptosis might constitute normal adjacent muscular contractions of skeletal or mimic muscle origin, externally applied force and gravity. In the face and neck region, the proposed function might contribute to protracted maintainment of soft tissue shape and volume.

## Testing of the hypothesis

Messenger RNA expression relevant to adipocyte apoptosis, determined in samples of human adipose cells obtained in connection with muscle activity in a physiological range, will constitute the basis for the verification. Samples of tissue fluid obtained by means of microdialysis and assessed with regard to triglycerides might yield additional information [5,14]. A measurable adiposity in the face and/or neck might be exposed to defined movement – the proposed end-point being local adipose volume reduction. A volumetric study of the facial adipose tissue volume in Bell's palsy patients by means of magnetic resonance imaging might also be informative.

## Implications of the hypothesis

The interpretation of metabolic disturbances by means of volumetric measurement of adipose tissue might to some extent be influenced.

In particular head and neck patients with deforming subcutaneous protrusions in surgical flaps might benefit from eliminating such deformities non-invasively by means of a mechanical device exposing the deforming tissue to controlled movement. The procedure might be repeated if local deformity should recur with eventual increase in body weight. Surgical alternatives would unavoidably cause new and at times unpredictable scar formation.

Face and neck excercise might find a place as non-invasive prophylaxis or treatment of cosmetic adipose deformity.

## Competing interests

The author(s) declare that they have no competing interests.
